# Experimental study on preparation and anti-tumor efficiency of nanoparticles targeting M2 macrophages

**DOI:** 10.1080/10717544.2021.1921076

**Published:** 2021-05-14

**Authors:** Zheng Zeng, Yu Liu, Qinglian Wen, Yixian Li, Jing Yu, Qiang Xu, Wenwu Wan, Yu He, Chen Ma, Yan Huang, Helin Yang, Ou Jiang, Fuyu Li

**Affiliations:** a Department of Oncology, The Affiliated Hospital of Southwest Medical University, Luzhou, China; b Department of Surgical Oncology, The Second People's Hospital of Neijiang, Neijiang, China; c Clinical Medical College of Southwest Medical University, Luzhou, China; d Department of Oncology, The Fourth People's Hospital of Neijiang, Neijiang, China; e Department of Oncology, The First People's Hospital of Neijiang, Neijiang, China; f Department of Biliary Surgery, West China Hospital of Sichuan University, Chengdu, China

**Keywords:** Mannose, imidazole, carboxymethyl chitosan, nanoparticle, M2 macrophages, malignant tumor

## Abstract

This study aimed to develop an effective therapy against M2 macrophages and to investigate the effects of imidazole and mannose modified carboxymethyl chitosan-nanoparticles (MIC-NPs) on tumor growth and antitumor immune responses. MIC-NPs were constructed and analyzed through ^1^H NMR, nano-laser particle size analyzer, and transmission electron microscopy. The nanoparticles were mainly distributed in 75–85 nm, and zeta potential was 1.5 mV. Cytotoxicity studies *in vitro* and *in vivo* indicated that MIC-NPs were safe. The targeting effect of MIC-NPs on M2 macrophages was observed through fluorescence microscope and microplate system. The results demonstrated the uptake of a large amount of FITC-loaded MIC-NPs by M2. Cell growth inhibition experiments showed that MIC-NPs significantly inhibited M2 through cell apoptosis. The evaluation of anti-tumor activity *in vivo* showed that MIC-NPs could accumulate in the tumor site to exert an anti-tumor effect. Flow cytometry showed that the proportion of M2 macrophages at the tumor site in the experimental group was significantly lower than that in the control group, while the Treg cells and cytotoxic T cells (CTL) were found to be increased. PCR detection showed that the cDNA of FIZZ, MR, TGF-β, and arginase, closely related to M2 macrophages, in the experimental group, was significantly lower than that in the control group, but there was no significant difference in the cDNA of Treg cell characteristic Foxp3 between the two groups. These results suggest that MIC-NPs are expected to provide a new and effective treatment for tumor.

## 1. Introduction

Conventional therapy, such as radiotherapy and chemotherapy, kills malignant cells directly. Due to the genetic instability, malignant cells tend to accumulate mutation, which can lead to the eventual failure of conventional therapy. Different from conventional therapy, immunotherapy such as the use of immune checkpoint inhibitors has a more lasting effect (Garon et al., 2019).

Under the pressure of immune selection, malignant cells evolve constantly, evade immune killing, recruit a variety of cells to help themself resist immune cell attack, gain the ability of infiltration, metastasis, and angiogenesis, and eventually develop from carcinoma *in situ* to invasive carcinoma (Emens, 2010; Wynn et al., 2013; Mascaux et al., 2019; Barrueto et al., 2020; Zins & Abraham, 2020). Hence, the immune cells in the tumor microenvironment (TME),play an important role in growth of the tumor, invasion, and metastasis (Feng et al., 2010; Welford et al., 2011; Stephen & Hajjar, 2020). Therefore, immunotherapy, which targets the immune cell in the TME, has become an alternative and new therapeutic approach and it has achieved certain success (Hu et al., 2020; Zhou et al., 2020). Due to the genetic stability, mutational rate in immune cell is very low, therefore, immunotherapy for cancer is expected to have a lasting effect, compared with radiotherapy and chemotherapy (Garon et al., 2019).

The current immunotherapy is mainly aimed at improving the anti-tumor function of cytotoxic T cells (CTL), but macrophages are the main and key components in TME, accounting for 50–80% of solid tumor cells (Lewis & Pollard, 2006; Solinas et al., 2010; Guerrouahen et al., 2019). The macrophages in the tumor lesions are characterized by M2 polarization, which is usually named as M2 macrophages (M2) (Lawrence & Natoli, 2011; Aras & Zaidi, 2017; Jayasingam et al., 2019). Different from the anti-tumor properties of M1 macrophages (M1), M2 plays an extremely important role in many key links such as the occurrence and development of tumor. For example, it promotes the formation of neoangiogenesis, inhibits the activation of T-cells, and enhances the invasive ability of tumor (Galdiero et al., 2013; Leifler et al., 2013; Klingen et al., 2017; van Dalen et al., 2018; Lin et al., 2019). Therefore, targeted regulation of M2 may become a new immunotherapy strategy for malignant tumor (Zheng et al., 2018; Li et al., 2019; Park, 2019; Zhang et al., 2020).

A remarkable feature of M2 in TME is that it has a number of mannose receptors (MRs), which is distributed throughout its cytomembrane (Gan et al., 2019). MR receptor belongs to the C-lectin family, and expressed mainly on the M2 of the human body. Mannose is a six-carbon monosaccharide that can specifically bind to the MR on the surface of M2 (Yeeprae et al., 2006). Yeeprae et al. found that mannosylated (Man) emulsions with 5% mannosylated substitution are sufficient for the recognition and endocytosis of macrophages via MR-mediated mechanism, thus mannosylated complexes are highly suitable for gene delivery of macrophages (Kawakami et al., 2000; Glass et al., 2019). Studies have confirmed that mannosylated macromolecular organics (such as liposomes) and mannosylated macromolecular materials (such as polylysine) can successfully deliver genes to dendritic cells and macrophages for the treatment of inflammation and tumor (Diebold et al., 2002; de Leoz et al., 2011).

Imidazole group can aggregate isopentene pyrophosphate by inhibiting -ferric pyrophosphate synthase and thereby it can lead to cell apoptosis (Gu et al., 2015). Imidazole-based drugs possess remarkable anticancer activities, due to their ability to hinder cell growth and cell division. It is an important part of the molecular structure of many drugs including anti-tumor drugs, dacarbazine, temozolomide, zoledronic acid, mercaptopurine, nilotinib, tipifarnib, etc., which has been reported for the effect of cytotoxicity and anti-cancer properties (Ali et al., 2017). As a kind of synthetic pyrophosphate in which the basic molecular structure is ‘P-C-P’, etidronic acid cannot be metabolized by biological cell and then accumulate in the cell after entering cell. Pyrophosphate can inhibit the activity of some enzymes of cells, especially farnesyl pyrophosphate synthase, and lead to cell apoptosis (Brown & Holen, 2009).

In this study, we intend to prepare a kind of nanoparticles which are assembled by ion cross-linking between imidazole and mannose-modified carboxymethyl chitosan and etidronic acid, observe its ability to target M2 of TME and apoptosis-inducing to M2, and see if it can control malignant tumor in animal models. The study might be paying the way for further research of the nanoparticles *in vivo*.

## 2. Experimental methods

### 2.1. Preparation and characterization of MIC-NPs

The MAN and O-CMC solutions of 10 mg/mL were obtained by adding α-d-mannose-4-aminophenyl glycoside (MAN) and carboxymethyl chitosan (O-CMC) to morpholine ethane sulfonic acid (MES) buffer, respectively. Hundred milliliters of MAN solution was mixed with 100 mL of O-CMC solution. 1-(3-Dimethylaminopropyl)-3-ethyl carbodiimide hydrochloride (EDC) 704 mg and N-hydroxy succinimide (NHS) 70 mg were added to the mixture and stirred for 12 h at 95 °C. Eventually, the obtained white powder was dissolved in 100 mL MES buffer. 1-(3-Aminopropyl) imidazole (N3A1) was added to the MES buffer to obtain 10 mg/mL N3A1 solution.

The above two solutions were mixed with 100 mL. Around, 1528 mg EDC and 153 mg NHS were added to the mixture and stirred at 30 °C for 12 h. A white powder was obtained by filtering and drying. The white powder is imidazole and mannose modified carboxymethyl chitosan (MIC). The production process of MIC is shown inScheme 1.

**Figure 1.**
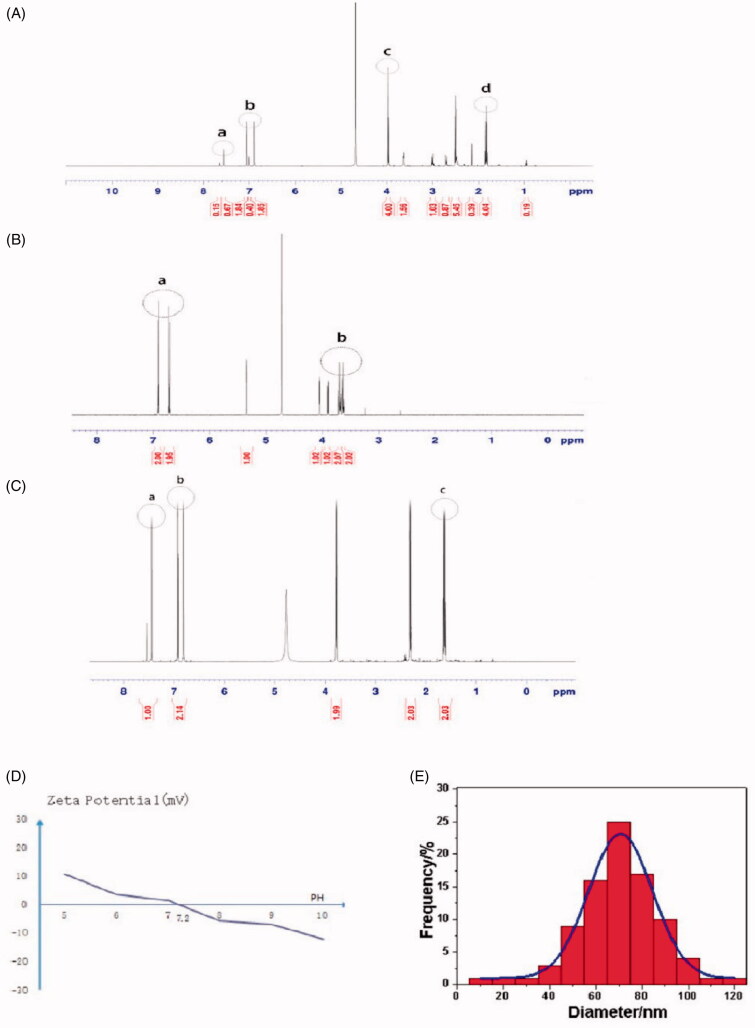
(A) ^1^H NMR spectra of MIC-NPs. (B) ^1^H NMR spectra of NDM. (C) ^1^H NMR spectra of N3AI. (D) Zeta potential of nanoparticles. (E) Nanometer particle size distribution. MIC-NPs: nanoparticles of imidazole and mannose modified carboxymethyl chitosan; NDM: P-aminophenol-d-pyran mannoside; N3AI: 1-(3-aminopropyl) imidazole.

MIC and etidronic acid were dissolved in distilled water to obtain 4 mg/mL MIC solution and 1 mg/mL etidronic acid solution, respectively. The MIC solution was dripped with glacial acetic acid to adjust the pH to 6. The MIC solution of 20 mL was stirred by 200 r/minutes at room temperature for 15 minutes, and the etidronic acid solution was added according to the MIC:disodium etidronate mass ratio of 1:1. The resulting mixture was then placed in a dialysis bag (MWCO: 3.5 kDa, Spectrum Laboratories, Los Angeles, CA) for 24 h, the product was freeze-dried in vacuum at −80 °C to obtain nanoparticle powder. The MIC-NPs were obtained by passing through the column in protein purification system (GE, AKTA Purifier, Uppsala, Sweden). The chemical structure of MIC-NPs is shown inScheme 1.

**Figure 2.**
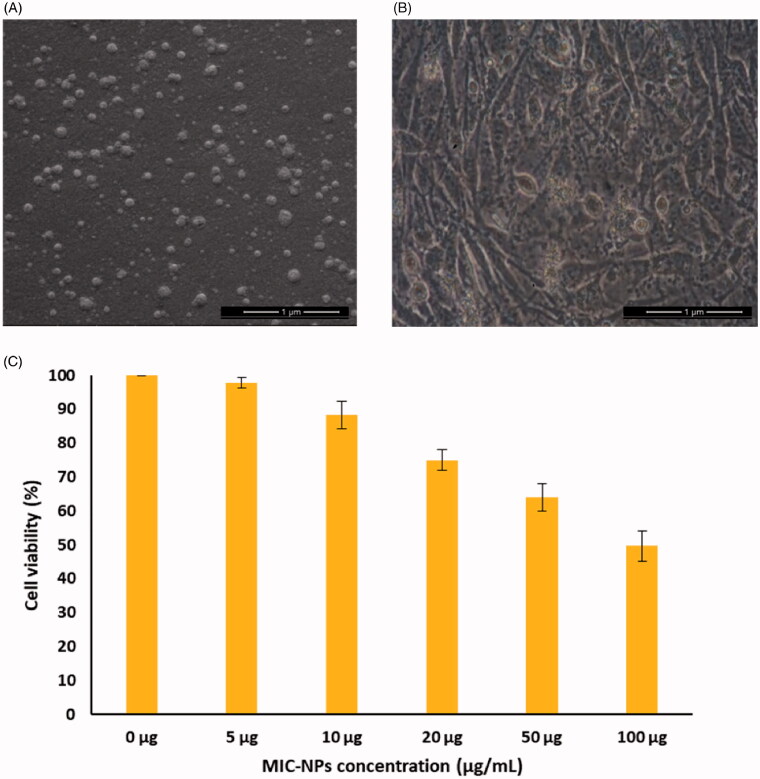
(A) The morphological image of MIC-NPs. (B) Human fibroblasts after adding MIC-NPs. (C) Effect of MIC-NPs on cell viability.

The samples were determined by proton nuclear magnetic resonance (^1^H NMR) dissolved in dimethyl sulfoxide-d6 at 600 Hz (Bruker ACF600, Billerica, MA). The average particle size and zeta potential of the MIC-NPs were measured by dynamic light scattering (DLS; NanoBrook 90Plus Zeta, Brookhaven, NY) at 25 °C. The morphological characteristics of the MIC-NPs were examined by transmission electron microscopy (Tecnai G2 F20, Hillsboro, OR).

### 2.2. *In vitro* cytotoxicity studies

Human fibroblasts (ShenZhen Otwo Biotechnology Co. Ltd., Shenzhen, China) were divided into experimental group and control group. MIC-NPs at a concentration range of 20 μg/mL were added in the experimental group and the same amount of PBS was added in the control group. Human fibroblast cells were seeded in 96-well plates at a density of 5 × 10^4^ cells per well, and subsequently incubated in hepatocyte maintenance medium (HMM) at 37 °C under 5% CO_2_ for 24 h. The toxicity of the drug to cells was determined by CCK-8 reagent method (Shanghai Biyuntian Biological Reagent Co. Ltd., Shanghai, China). According to the instructions of the reagent, the absorbance (OD) at 450 nm was detected using a microplate reader (Bio-Rad, Hercules, CA). The inhibition rate was calculated by collecting data at 12 h, 24 h, 48 h, and 72 h after the addition of nanoparticles, relative growth rate (RGR)=(1 – experimental group OD/control group OD)×100%. The cytotoxicity was evaluated according to the toxicity grading method of American Pharmacopoeia (USP XXII, NF XVII [S]; USP, 1990).

### 2.3. Induction of M1 and M2 macrophages

U937 cells (ShenZhen Otwo Biotechnology Co. Ltd., Shenzhen, China) were induced into M1 by IFN-γ and LPS, and M2 by IL-4 and IL-13 (Vats et al., 2006; Odegaard et al., 2007). M1 macrophages express high-level of inducible nitric oxide synthase (iNOS) and low-level ion of arginase 1 (Arg-1) while, M2 expresses low level of iNOS and high level of CD206 (Yeeprae et al., 2006). Therefore, flow cytometry (FCM) (BD FACSVerse, BD Biosciences, San Jose, CA) was used to detect iNOS and CD206 to determine whether U937 was successfully induced into M1 or M2 macrophages.

### 2.4. Cellular uptake study and mannose receptor blocking study

Cellular uptake was evaluated by fluorescence microscope (Olympus, Tokyo, Japan) and microplate system (CORONA, Tokyo, Japan) with FITC as a fluorescent marker. U937, M1, and M2 cells were seeded in six-well plates at a density of 10^6^ cells per well for 24 h. Dil at a concentration range of 10 μL/mL was added to these cells and stained for 10 minutes. Then, FITC-loaded MIC-NPs were added in the experimental group, and unloaded MIC-NPs were added in the control group. The mixtures were incubated in incubator at 37 °C under 5% CO_2_ for 3 h. One milliliter of RPMI medium (1640, Gibco, Waltham, MA) was added and the cells were further cultured for 2 h. After washing with PBS (0.01 M, pH 7.4) for a total of three times, the cells were analyzed by fluorescence microscope and microplate system. The relative fluorescence intensity (RFU) was used to indicate the uptake of MIC-NPs by these cells. RFU = fluorescence intensity in experimental group/fluorescence intensity in control group.

M2 cells were seeded in six-well plates at a density of 10^6^ cells per well for 24 h. One microliter of mannose was added in the experimental group; 3 h later, FITC-loaded MIC-NPs were added in the experimental group, and unloaded MIC-NPs were added in the control group. The incubation method is the same as above. The cells were analyzed by fluorescence microscope and microplate system.

### 2.5. Mechanism analysis of MIC-NPs inhibiting M2

Apoptosis mainly depends on two pathways: mitochondrial dependence and death receptor, the two activation pathways will eventually merge and complete the apoptosis process at caspase-3 (Xia et al., 2020). Therefore, we used caspase-3 (Shanghai Biyuntian Biological Reagent Co., Ltd., Shanghai, China) to detect M2 apoptosis. The procedure was carried out according to the manufacturer’s instructions. Then, the cell apoptosis was measured at 0 h, 24 h, 48 h, and 72 h after the nanoparticles were added. The morphological characteristics of the cells were examined by transmission electron microscopy and the cell apoptosis was analyzed and recorded at 450 nm using a microplate reader (Bio-Rad, Hercules, CA).

Meanwhile, we carried out the detection of pyrophosphonate nitrite synthase (FPPs) according to the manufacturer’s instructions. M2 cells were seeded in six-well plates at a density of 10^6^ cells per well for 24 h. Ten nanograms per milliliter of MIC-NPs was added in the experimental group and PBS was added in the control group. Then, cellular proteins were extracted at 12 h, 24 h, and 48 h. Western blot was used to detect FFPs content in total protein. Finally, the absorbance value of protein was analyzed by Gel Image processing system (Bio-Rad, Hercules, CA).

### 2.6. Cell growth inhibition study

The growth inhibition of cells by MIC-NPs was evaluated using a CCK-8 assay. M1, M2, and Lewis (ShenZhen Otwo Biotechnology Co. Ltd., Shenzhen, China) were used as model cells. M1, M2, and Lewis cells were seeded in 96-well plates with a density of 5 × 10^4^ cells per well. Subsequently, 100 μL of HMM and MIC-NPs at a concentration range of 10 ng/μL were individually added. Then, the mixtures were incubated in incubator at 37 °C under 5% CO_2_ for 12 h. At determined time points, 10 μL of CCK-8 was added and the cells were then incubated for an additional 1 h. Then, the cell proliferation was measured at 12 h, 24 h, 48 h, and 72 h. The cell growth inhibition was analyzed and recorded at 490 nm using a microplate reader (Bio-Rad, Hercules, CA). Each treatment condition was evaluated in triplicates in three independent experiments.

### 2.7. Toxicity test *in vivo*

C57 mice (male, 6 weeks old, weight 20 ± 5 g) were provided by the Animal Center of Southwest Medical University (Luzhou, China). All the animal experiments were conducted strictly according to the Guiding Principles for the Use of Laboratory Animals and were approved by the Institutional Animal Care and Use Committee of Southwest Medical University.

A total of 16 C57 mice were treated with MIC-NPs. These mice were randomly divided into four experimental groups, which received the following doses: 5 mg/kg, 50 mg/kg, 500 mg/kg, and 2000 mg/kg. Four C57 mice treated with 0.4 mL of saline solution served as control group. The drug was administered via tail vein injection. The heart, liver, spleen, lung, and kidney were dissected and weighed after treating with drugs.

### 2.8. Pharmacokinetics

Lewis xenografts were established by subcutaneous injection of a suspension of Lewis cells (5.0 × 10^6^ cells/mL in 0.2 mL PBS) into the right leg region of nude mice. When the tumor reached a diameter of 100–300 mm^3^, these tumor-bearing nude mice were randomly divided into two groups of four animals each: normal saline (NS) group; MIC-NPs (at a standard dose of 250 mg/kg) group. At 3 h and 12 h after injection of FITC-loaded MIC-NPs, the mice were sacrificed and the hearts, livers, spleens, lungs, kidneys, and tumor were taken out and observed for live imaging system (excitation wavelength: 490 nm, emission wavelength: 520 nm) (IVIS SPECTRUM, Alameda, CA).

### 2.9. Anti-tumor efficacy of MIC-NP *in vivo*

The establishment of Lewis xenografts was the same as above. The mice were randomly divided into five groups of 10 animals each: group 1: saline (NS); group 2: MIC-NPs (250 mg/kg) and NS; group 3: MIC-NPs (500 mg/kg) and NS; group 4: MIC-NPs (1000 mg/kg) and NS; group 5: 500 μg of interferon. Group 1 represents the negative control group. Groups 2–4 represent the low, medium, and high dose experimental groups, respectively. Group 5 represents the positive control group. The mice were injected intravenously (via tail vein injection) twice a week. The tumor volume and the body weight of mice were measured twice a week. The tumor volume was calculated using the following formula:

$$V({\text{mm}}^{3})=0.5a\times b^{2}$$

where *a* represents the length and *b* represents the width of the tumor. According to the results, the relative tumor volume was calculated by RT=*V*_1_/*V*_0_. Among them, *V*_0_ was the tumor volume measured before the time of injection and *V*_1_ was the tumor volume at each measurement after injection. The evaluation index of antineoplastic activity was the relative tumor proliferation rate T/C (%):

$$T/C=\frac{\mathrm{TRT}}{\mathrm{CRT}}\times 100\%$$

where TRT is the experimental group RT; CRT is the negative control group RT.

### 2.10. Detection of immune cells by flow cytometry

In this study, the animals in group 1 and group 3 were sacrificed on day 21. Tumor tissue specimens were extracted from nude mice, cut into pieces, and immediately mixed with PBS (0.01 M, pH 7.4). The mixture was filtered using a 300 μm nylon net to harvest tumor cells. Then, the suspension was washed twice with PBS (0.01 M, pH 7.4) after centrifugation (800–1000 r/minutes, five minutes). The precipitate was then resuspended in 1 mL of NS. Fluorescent antibodies were added to the suspension at 4 °C for 15 minutes and then analyzed by FCM. Flow cytometry was used to detect CD206, iNOS, F4/80, CD8, CD28, CD4, and Foxp3. These markers are mainly targeted at M2, CTL, and Treg immune cells.

### 2.11. Quantitative real-time PCR detection

In the animal model of Lewis lung cancer in the above design, the animals in group 1 and group 3 were killed on the 21st day, and the lungs of the mice were dissected.

#### 2.11.1. Extraction of RNA

The lung tissue was grounded into powder by grinding with liquid nitrogen, followed by blowing with 1 mL Trizol. The samples were collected in the 1.5 mL RNA enzyme free EP tube. To that, 0.2 mL of chloroform was added, shaken violently for 15–20 seconds and mixed evenly. The samples were allowed to stand at room temperature for five minutes followed by centrifugation at 12,000 r/minutes for 10 minutes at 4 °C and upper liquid is carefully transferred to the new EP tube. An equal volume of isopropanol was added (0.4 mL). The samples were mixed thoroughly and incubated at room temperature for 10 minutes. Then, the tubes were centrifuged at 4 °C (12,000 r/minutes) for 10 minutes and the resulting supernatant was discarded. Around, 1 mL of 75% ethanol was added and centrifuged at 4 °C for five minutes. The supernatant liquid was carefully suck out with a sample gun. The samples were allowed to air dry until the pellet becomes transparency. After drying, an appropriate amount of DEPC was added to the tube to dissolve the RNA. The concentration of RNA in the sample was quantified. The samples were stored at −70 °C refrigerator until further use.

#### 2.11.2. Detection of RNA integrity

The electrophoretic techniques were used to judge the integrity of RNA.

#### 2.11.3. Reverse transcription

Reverse transcription was performed according to the instructions given in reverse transcription kit. The quantity of RNA needed for each 20 μL reaction system is 900 ng. The volume of RNA solution needed for reverse transcription is calculated according to the concentration of RNA. The genomic DNA contamination is removed prior to reverse transcription process. 

**Table:** 

Composition	Sample volume
5 × gDNA eraser buffer	2.0 μL
gDNA eraser	1.0 μL
RNA	Calculated volume *x* based on concentration
RNase free dH20	7 – *x*

After mixing and centrifugation, the ordinary PCR instrument was used at 42 °C for two minutes and 4 °C for five seconds. The reverse transcription reaction was added on the ice, and the liquid matching system was as follows: 

**Table:** 

Composition	Sample volume
5× PrimeScript buffer	4.0 μL
PrimeScript RT enzyme MIX 1	1.0 μL
RT Primer MIX	1.0 μL
The reaction solution of the second step	10 μL
RNase free dH20	4.0 μL

After mixing and centrifugation, reverse transcription was carried out on an ordinary PCR instrument, and the conditions were as follows: 37 °C for 15 minutes, 85 °C for five seconds, and 4 °C for five minutes. The samples were stored at −20 °C for use.

#### 2.11.4. Real-time fluorescence PCR analysis

Real-time fluorescence quantitative PCR detection set the reaction system as 20 μL. To operate on ice, the reaction system was set up according to the instructions, mixed gently and centrifuged. Real-time PCR conditions are given as follows. 

**Table:** 

95 °C	5 s
95 °C	5 s
60 °C	30 s

A total of 45 cycles were performed. Three multiple holes were set up in the experiment of fluorescent PCR to avoid the influence of accidental results.

#### 2.11.5. Primer sequence

**Table:** 

Gene	5′–3′	5′–3′
Foxp-3	CTCATGATAGTGCCTGTGTCCTCAA	AGGGCCAGCATAGGTGCAAG
TGF-B	AACATGATCGTGCGCTCTGCAAGTGCAGC	AAGGAATAGTGCAGACAGGCAGGA
Arginase	CAGAAGAATGGAAGAGTCAG	GGTGACTCCCTGCATATCTG
MR	CATGAGGCTTCTCTTGCTTCTG	TTGCCGTCTGAACTGAGATGG
FIZZ1	GGTCCCAGTGCATATGGATGAGACC	CACCTCTTCACTCGAGGGACAGTTG
GAPDH	TGTAGACCATGTAGTTGAGGTCA	AGGTCGGTGTGAACGGATTTG

### 2.12. Statistical analysis

Statistical analysis was carried out using SPSS 19.0 software (SPSS, Chicago, IL). The difference of experimental data was analyzed by independent *t*-test or one-way ANOVA test. Data were shown as mean ± standard error. The means were determined to be different with *p*<.05 and deemed significantly different with *p*<.01.

## 3. Results

### 3.1. Characterization of nanoparticles

The chemical structure of the nanoparticles was confirmed by ^1^H NMR. As shown in the ^1^H NMR spectra of MIC-NPs, the MIC-NPs showed four characteristic hydrogen peaks, a, b, c, and d (Figure 1(A)). The ^1^H NMR spectra of NDM are shown in Figure 1(B). The imidazole groups and hydrocarbon chain of N3AI units showed their characteristic peak a (*δ* = 7.57 ppm) and peak d (*δ* = 1.80–1.85 ppm), the overlapping hydrogen atoms from benzene ring of NDM units and imidazole group of N3AI units were peak b (*δ* = 6.90–7.06 ppm), the overlapping hydrogen atoms from hydrocarbon chain on N3AI and NDM were peak c (*δ* = 3.95–3.98 ppm), which confirmed the successful synthesis of MIC-NPs.

The area ratio of peak b and peak a of MIC-NPs is 5.68. The area ratio of peak b and peak a of N3AI is 2.14 (Figure 1(C)). Therefore, it indicated that the hydrogen atom in MIC-NPs is one time more than that in N3AI, which all suggested the formation of MIC-NPs.

The zeta potential of MIC-NPs detected by DLS was 1.5 mV at pH = 7 and 0 mV at pH = 7.2 (Figure 1(D)), with a narrow range of particle size distribution of 77.2 ± 13.5 nm (Figure 1(E)). The average diameter of the nanoparticles was 77 nm. The morphology of the nanoparticles was observed by electron microscopy. The nanoparticles were spherical in shape, with proper sizes, and were found to disperse well in aqueous solution.

### 3.2. *In vitro* cytotoxicity analysis

Through the observation of electron microscope, it can be seen that the morphology of human fibroblasts treated with nano-drug MIC-NPs is basically normal, and the cells adhere to the wall in a fusiform shape (Figure 2(A,B)). CCK-8 assays were conducted in order to investigate the cytotoxicity of MIC-NPs *in vitro*. Table 1 shows the cytotoxic level of MIC-NPs on human fibroblasts. This finding indicated that the MIC-NPs copolymer exhibited a slight cytotoxicity. However, the higher concentration of MIC-NPs exhibited the lower cell viability (Figure 2(C)). Therefore, MIC-NPs were considered as safe nanoparticles *in vivo*.

**Table 1.** Cytotoxicity of nanoparticles toward human fibroblasts.

Group	12 h		24 h		48 h		72 h	
	RGR (%)	Grade	RGR (%)	Grade	RGR (%)	Grade	RGR (%)	Grade
Experimental	122	0	75.8	0	75.5	0	84.7	0
Control	100	0	100	0	100	0	100	0

#### 3.2.1. Induction of macrophages

**Figure 3.**
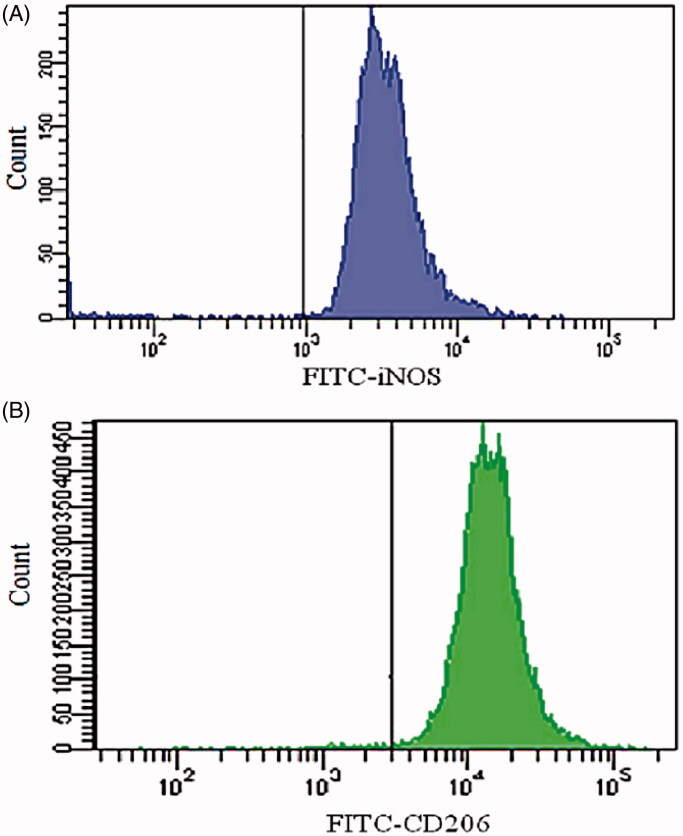
(A) iNOS was mainly expressed in cells induced by IFN-γ and LPS. (B) CD206 was mainly expressed in cells induced by IL-4 and IL-13. RGF: relative growth rate; iNOS: inducible nitric oxide synthase; IFN-γ: interferon-γ; LPS: lipopolysaccharide; IL-4: interleukin-4; IL-6: interleukin-6.

Flow cytometric analysis was used to evaluate the induction of macrophages. As shown in Figure 3(A), FCM showed that 91.6% of the cells expressed iNOS after U937 was stimulated with IFN-γ and LPS for 72 h, indicating that M1 macrophages were successfully induced. As shown in Figure 3(B), FCM showed that 96.4% of the cells expressed CD206 after U937 was co-stimulated by IL-4 and IL-13 for 72 h, indicating that M2 was successfully induced.

#### 3.2.2. Cellular uptake and mannose receptor blockade

**Figure 4.**
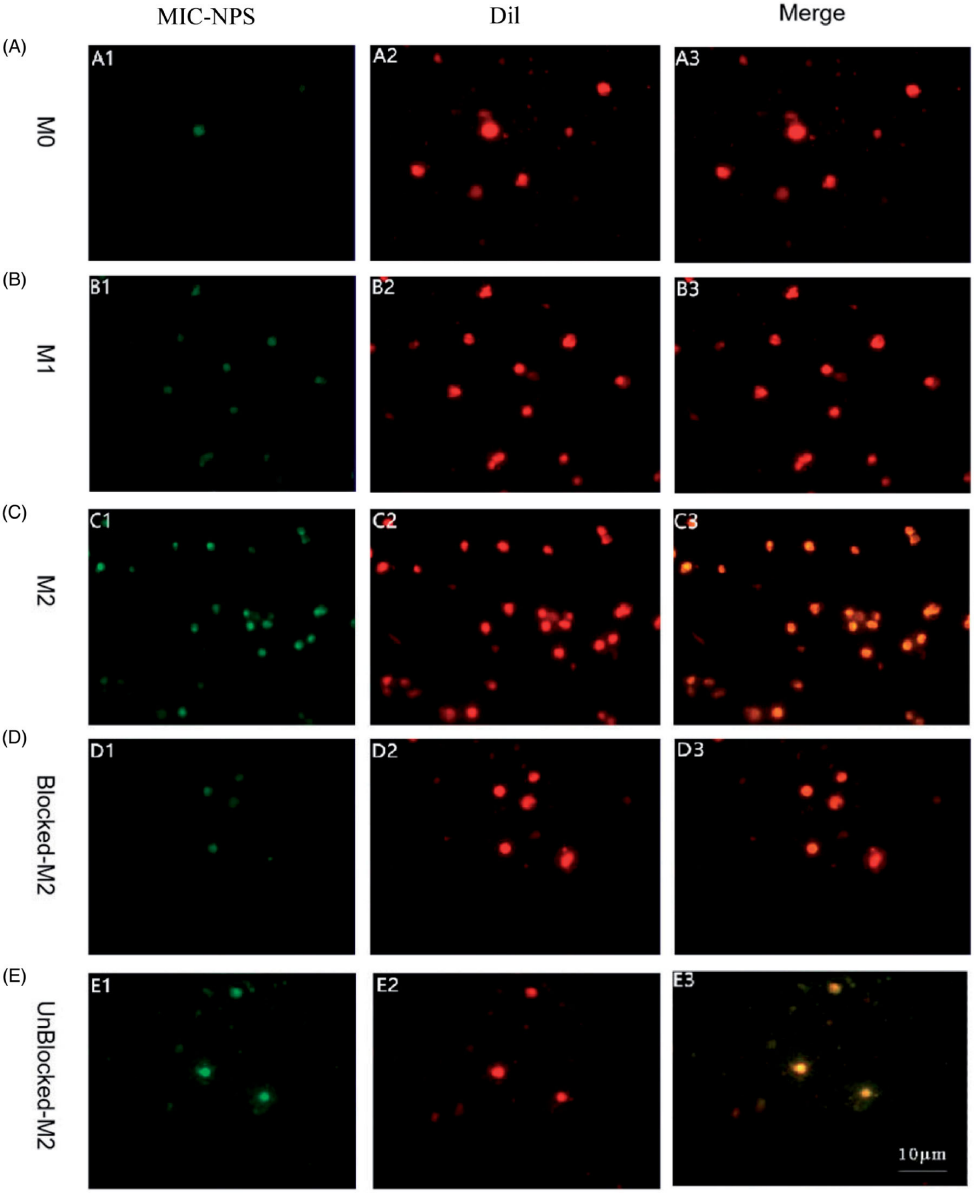
(A) Fluorescent images of M0 cells treated with FITC-loaded MIC-NPs and Dil. (B) Fluorescent images of M1 cells treated with FITC-loaded MIC-NPs and Dil. (C) Fluorescent images of M2 cells treated with FITC-loaded MIC-NPs and Dil. (D) Fluorescent images of M2 cells after blocking the mannose receptor. (E) Fluorescent images of M2 cells without blocking the mannose receptor. FITC-loaded MIC-NPs: nanoparticles of imidazole and mannose modified carboxymethyl chitosan loaded with FITC.

The cell membranes of M0, M1, and M2 were labeled with Dil and FITC labeling MIC-NPs. Dil shows red fluorescence after binding to cell membrane lipid, while FITC can combine with MIC-NPs to produce yellow-green fluorescence. Hence, the relationship between nano-drug MIC-NPs and cells can be clearly shown under fluorescence microscope. The cellular uptake in M0, M1, and M2 cells after incubation with FITC-loaded MIC-NPs and Dil are shown in Figure 4(A–C). No obvious fluorescence signal was observed in M0 and M1 upon treatment with FITC-loaded MIC-NPs. However, M2 showed remarkable fluorescence intensity in the FITC-loaded MIC-NPs, suggesting that M2 has specific cellular uptake characteristics to MIC-NPs.

The fluorescence intensity of M2 after incubation with MIC-NPs was significantly higher than the other two kinds of cells by microplate system (*p*<.01) (Table 2). The fluorescence intensity of M1 was higher than that of U937, but there was no obvious difference (*p*>.05). The results suggested that M2 could specifically uptake the nanoparticles compared with other cells.

**Table 2.** Fluorescence intensity and RFU value of each group.

	U937	M1	M2
Experimental group	123.7 ± 23.4	135.1 ± 17.9	873.8 ± 225.3
Control group	115.3 ± 14.5	111.7 ± 13.6	125.1 ± 19.4
RFU	1.08 ± 0.03	1.21 ± 0.02	6.84 ± 0.16

RFU: relative fluorescence intensity.

As shown in Figures 4(D) and 3(E), after the MR on the surface of M2 was blocked, the fluorescence intensity of M2 co-incubated with FITC-loaded MIC-NPs was significantly lower than that without blocking. The finding suggested that the binding of MIC-NPs to M2 was blocked by mannose to a certain extent.

#### 3.2.3. Cell growth inhibition study

**Figure 5.**
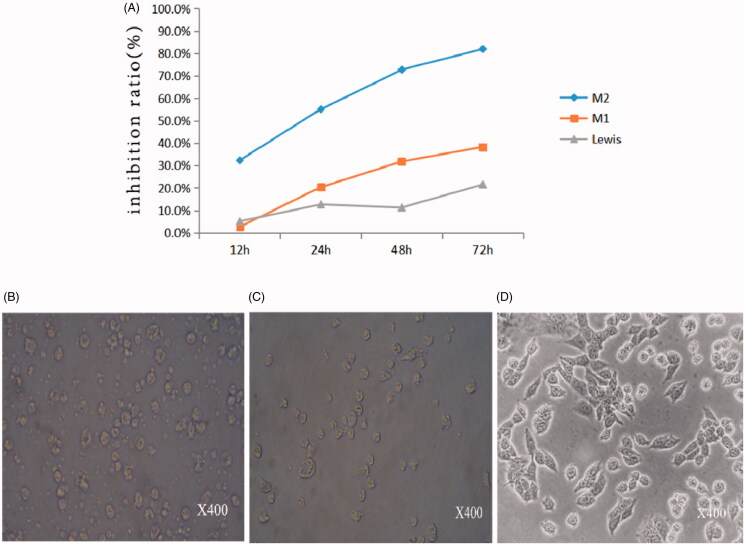
(A) The growth inhibition of MIC-NPs on three kinds of cells. (B) The morphology of M2 after incubation with MIC-NPs for 24 h. (C) The morphology of M1 after incubation with MIC-NPs for 24 h. (D) The morphology of Lewis cells after incubation with MIC-NPs for 24 h. MIC-NPs, nanoparticles of imidazole and mannose modified carboxymethyl chitosan.

The growth inhibition of cells by MIC-NPs was evaluated using a CCK-8 assay. Figure 5(A) shows all three types of cells were inhibited by MIC-NPs. However, the inhibition on M2 was particularly prominent. The inhibition rate of M2 which was significantly higher than that in other groups (*p*<.001), indicating that MIC-NPs could target the inhibition of M2.

#### 3.2.4. Apoptosis analysis

The morphology of M2, M1, Lewis cells after incubation with MIC-NPs for 24 h is shown in Figure 5(B–D). Compared with M1 and Lewis cells, the M2 cells were no longer adhering to the wall without pseudopodia. Meanwhile, the cells contract into a circle. All the changes were consistent with the characteristics of apoptosis. Then, the similar changes were observed at 48 h and 72 h.

As shown in Table 3, the enzyme activity of M2 increased significantly at 12 h and reached the peak at 48 h by detecting caspase-3. Compared to the control group, the activity of caspase-3 increased significantly at four time points. The results suggested that MIC-NPs could lead to the inactivation of M2 apoptosis by stimulating the endogenous apoptosis pathway of M2.

**Table 3.** The activity of caspase-3 test.

Data	0 h	24 h	48 h	72 h
Experimental	216 ± 18.4	428 ± 31.3	615 ± 44.6	587 ± 41.6
Control	221 ± 13.1	211 ± 27.5	276 ± 23.5	312 ± 35.7
*t*	1.265	9.896	15.377	11.342
*p*	.275	.001	.001	.000

**Figure 6.**
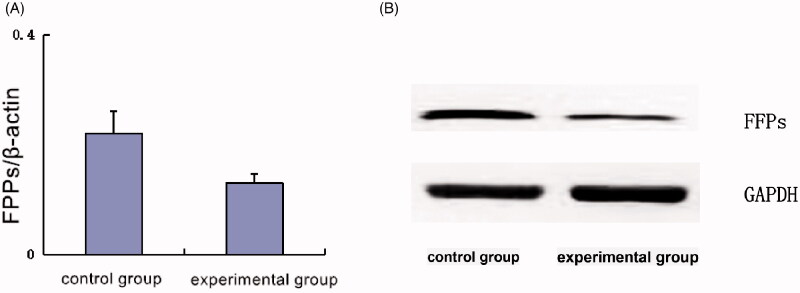
(A) Electrophoretic results of the two groups and (B) intracellular expression of FPPS in two groups.

By Western blot detection, the ratio of FPPs content in the experimental group was 0.13 ± 0.016, which was much lower than that in the control group (0.22 ± 0.04). It is suggested that MIC-NPs affect the expression of FPPs in M2 macrophages (Figure 6(A,B)).

### 3.3. Cytotoxicity analysis *in vivo*

**Figure 7.**
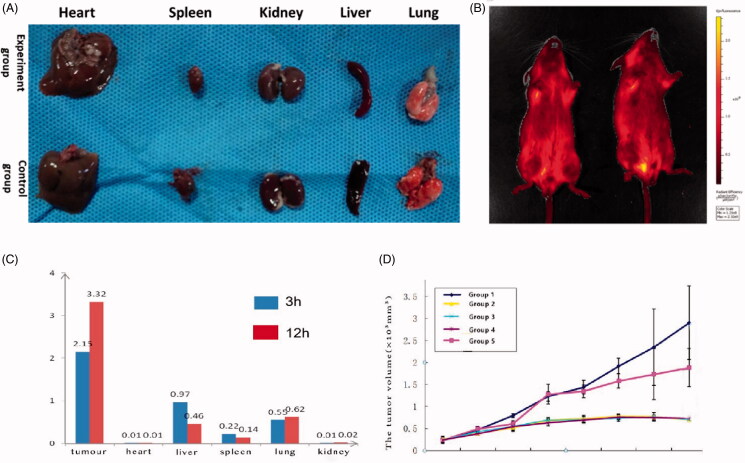
(A) The gross anatomical comparison of animal organs in two groups. (B) Fluorescent images of mice treated with FITC-loaded MIC-NPs. (C) Distribution of fluorescence intensity in tumor and organs in mice. (D) Tumor volume comparison between five groups (group 1: saline (NS); group 2: MIC-NPs (250 mg/kg) and NS; group 3: MIC-NPs (500 mg/kg) and NS; group 4: MIC-NPs (1000 mg/kg) and NS; group 5: 500 μg of interferon). FITC-loaded MIC-NPs: nanoparticles of imidazole and mannose modified carboxymethyl chitosan loaded with FITC.

Through the observation of toxicity experiment *in vivo*, we could see that no toxic reaction was observed within seven days. Besides, 10 mice were treated with 2000 mg/kg for 2 weeks. The results showed that there was no abnormal reaction in mice. As shown in Figure 7(A), no abnormalities were found in the visceral anatomy of the two groups. All the findings indicated that MIC-NPs were safe *in vivo*.

#### 3.3.1. Pharmacokinetics

Figure 7(B) shows the fluorescence imaging results *in vivo*, which more clearly demonstrated the distribution of nanoparticles in tumor, which suggested that MIC-NPs had preferable ability to target tumor site. Concentration of MIC-NPs was determined in various tissues of mice including heart, liver, spleen, lung, and kidney at 3 h and 12 h after a single dose intravenous administration of MIC-NPs as shown in Figure 7(C). FITC-load MIC-NPs showed fluorescence distribution in lungs, tumor, liver, and spleen at 3 h, accounting for 13%, 51%, 23%, and 11%, respectively, and there was only traced fluorescence in heart and kidney. At 12 h, the fluorescence intensity of liver and spleen decreased by 53% and 37%, respectively, and the fluorescence intensity of lung and tumor increased by 13% and 55%, respectively. The results indicated that MIC-NPs could accumulate in tumor sites obviously. The concentration of MIC-NPs in organs decreased with time while it increased in tumor sites.

#### 3.3.2. Anti-tumor effect in vivo

The anti-tumor efficacy of MIC-NPs was evaluated *in vivo* using Lewis cells xenografts in nude mice. The mice in the three experimental groups showed no abnormal reaction during the study, while the mice in the interferon group showed trembling, slow movement, and reduced food intake after injection, but recovered quickly. As shown in Figure 7(D), all experimental groups exhibited significant anti-tumor activity (*p*<.05), when compared with the NS group and interferon group. The relative tumor proliferation rates of MIC-NPs in the three experimental groups were similar (Table 4), and there was no significant difference in tumor volume (*p*<.05). Relative tumor proliferation rate was controlled after 12 days of administration. Relative tumor proliferation rate of the three experimental groups was less than 40% at 15 days. Then, the tumor volume did not increase significantly, and even showed a trend of shrinking. The results suggested that the MIC-NPs had anti-tumor effect to Lewis cells xenografts in nude mice. Meanwhile, the antitumor effect of MIC-NPs was not affected by dose.

**Table 4.** Results of relative tumor proliferation rate in different doses of nanoparticles.

T/C	2 d	5 d	8 d	12 d	15 d	18 d	21 d
Low	72.60%	74.00%	48.70%	40.80%	33.70%	29.50%	21.70%
Middle	85.10%	75.30%	53.40%	45.30%	35.90%	31.00%	23.00%
High	79.60%	77.00%	50.40%	46.40%	38.60%	31.10%	24.70%

#### 3.3.3. Detection of immune cells by flow cytometry

**Figure 8.**
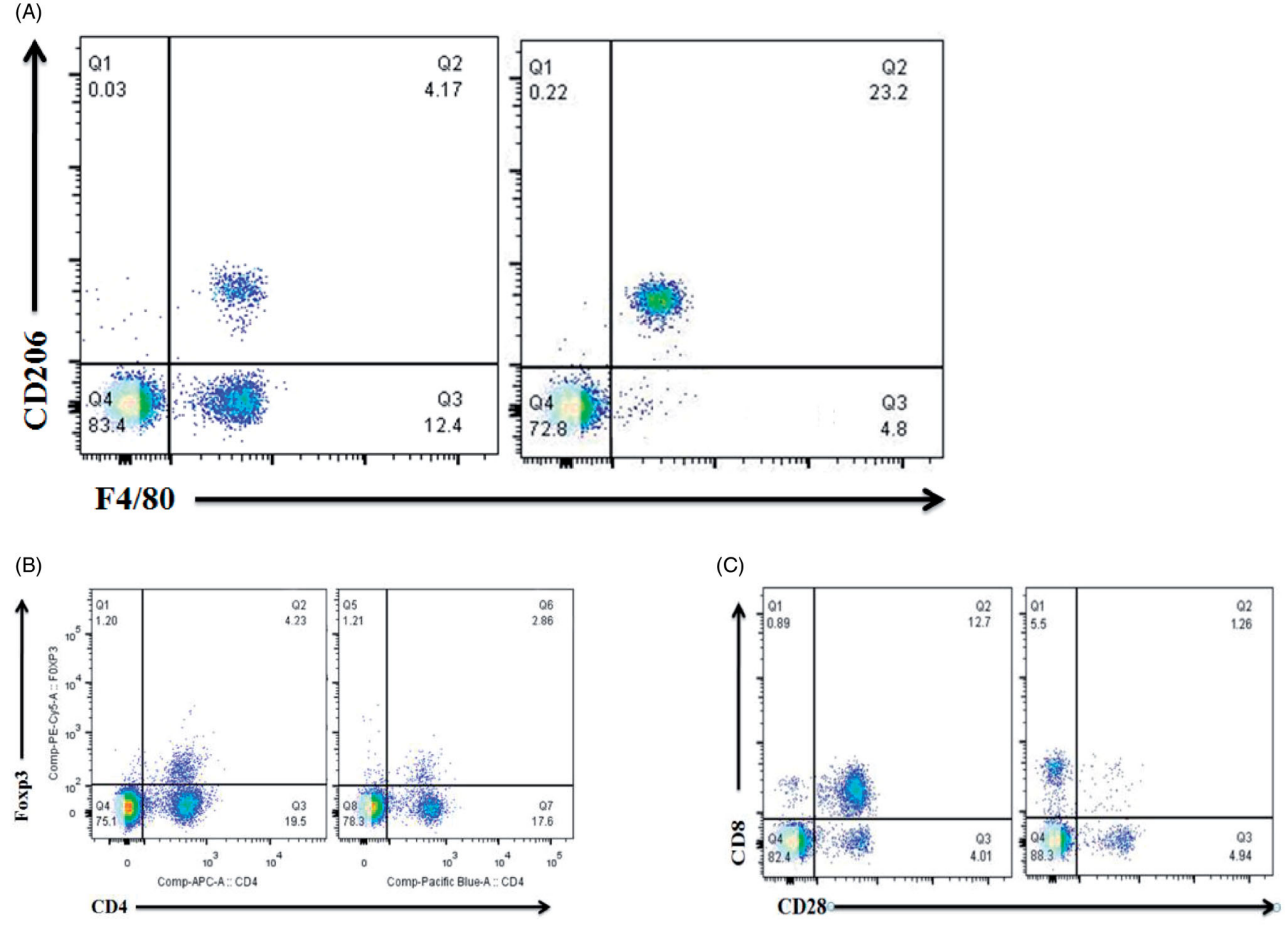
(A) Expression of CD206 and F4/80 in tumor site. (B) Expression of Foxp3 and CD4 in tumor site. (C) Expression of CD28 and CD8 in tumor site.

Immunohistochemical staining for CD206 and F4/80 was performed to assess the effect of M2 on tumor proliferation. As shown in Figure 8(A), the quantification of CD206 and F4/80 positive cells was lower in the MIC-NPs group (4.17%, *p*<.05) compared to that in the NS group (23.2%, *p*<.05). Immunohistochemical staining for CD4 and Foxp3 was performed to assess the effect of Treg on tumor proliferation. As shown in Figure 8(B), the quantification of CD4 and Foxp3 positive cells was higher in the MIC-NPs group (4.23%, *p*<.05) compared to that in the NS group (2.86%, *p*<.05). Immunohistochemical staining for CD28 and CD8 was performed to assess the effect of CTL on tumor proliferation. As shown in Figure 8(C), the quantification of CD28 and CD8 positive cells was higher in the MIC-NPs group (12.7%, *p*<.05) compared to that in the NS group (1.26%, *p*<.05). All the results showed that MIC-NPs could down-regulate M2 in tumor site and up-regulate the number of Treg cells and CTL, suggesting that MIC-NPs inhibited tumor proliferation by M2.

#### 3.3.4. PCR result

**Figure 9.**
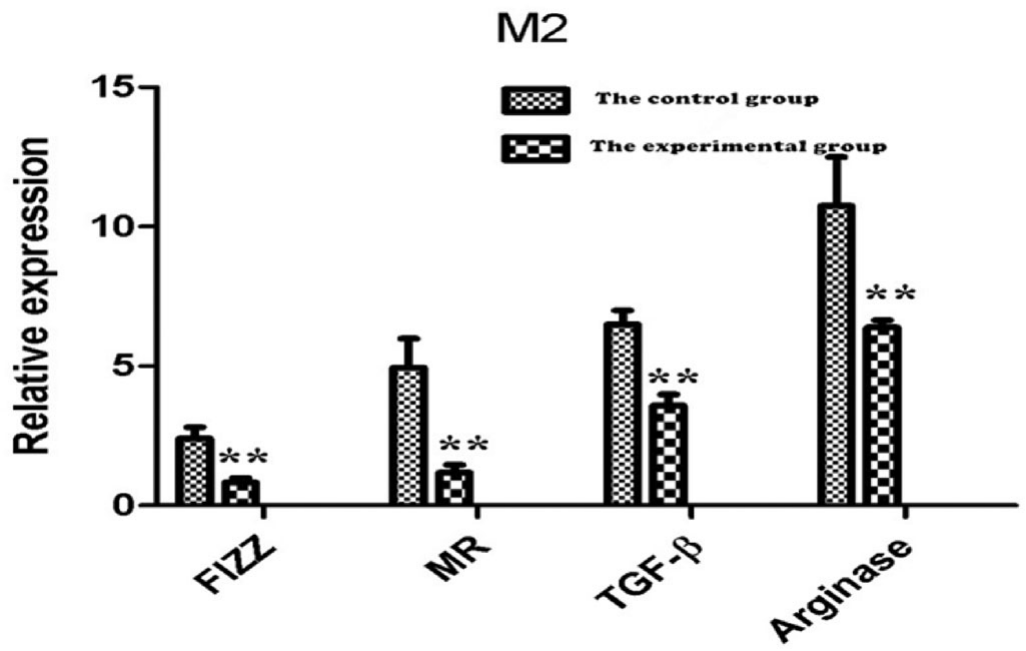
Comparison of cDNA relative expression values of FIZZ, MR, TGF-β, and arginase. The results of PCR-RT showed that the content of FIZZ/MR/TGF-B/arginase in the lung tissue of the experimental group was significantly lower than that of the control group (**p<.01).

**Figure 10.**
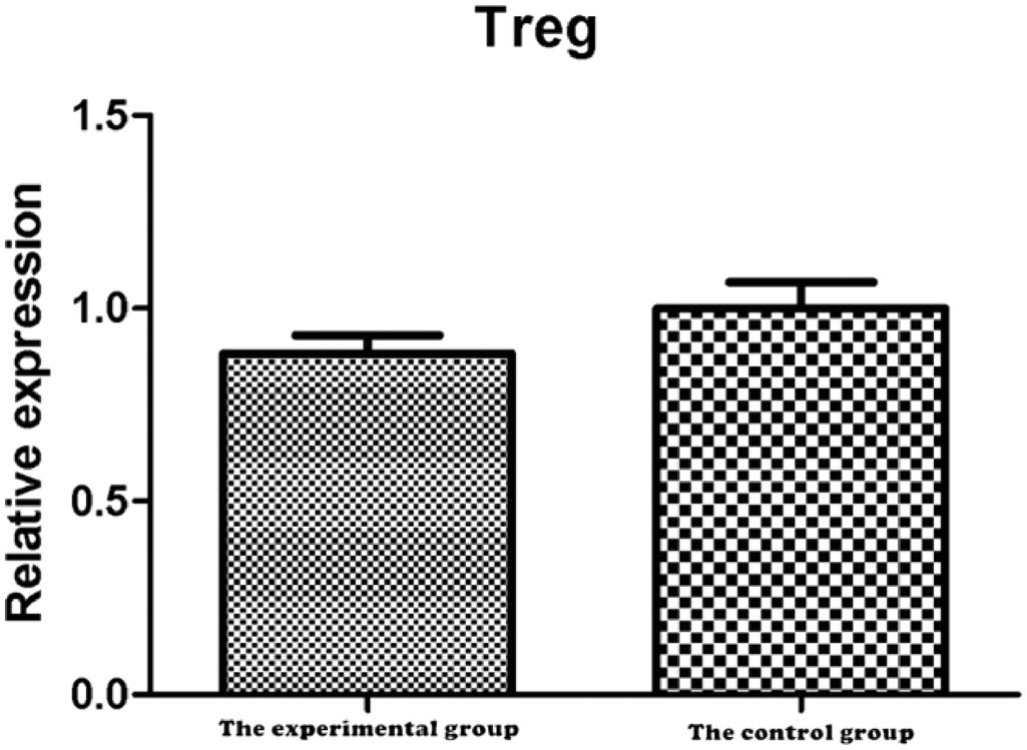
Comparison of cDNA relative expression values of Foxp3. The results of PCR showed that there was no significant difference in the content of Treg in the lung tissue between the experimental group and the tumor group (*t* = 0.943, *p*=.445>.05).

RNA was extracted from lung tissue for reverse transcription, and RT-PCR detection showed that the cDNA of FIZZ, MR, TGF-β, and arginase related to M2 cell densification in the experimental group were significantly lower than that in the control group (Figure 9), suggesting that the content of M2 in the lung tissue of the experimental group was significantly lower than that of the control group. However, there was no significant difference in the cDNA of Foxp3 characteristic of Treg cells between the two groups (Figure 10).

## 4. Discussion

Our research team successfully prepared MIC-NPs, a kind of mannosylated nanoparticles. Different from other cells, M2 swallows a mass of MIC-NPs and this phagocytosis effect was significantly weakened after blocking MR with mannose. The results suggested that MIC-NPs can specifically target M2 via MR-mediated mechanism. Our experimental results showed that inhibition rate of 10 μg/mL MIC-NPs (on M2) was 55.7% and 77.1% at 24 h and 48 h, respectively, which met the concentration requirements of antitumor drugs. However, MIC-NPs have no significant inhibitory effect on M1 and Lewis cells. These results indicate that the MIC-NPs not only specifically target the M2 but also lead to apoptosis of M2.

In aqueous solutions, the negatively charged phosphate group of etidronic acid can form intermolecular cross-linking with the positively charged amino group of carboxymethyl chitosan via ion cross-linking reaction, so nanoparticles with different particle sizes can be formed by controlling the reaction conditions (concentration ratio, mass ratio, pH value, etc.). On the other hand, solid tumor has the characteristics of high permeability and retention effect (i.e. increased vascular permeability and reduced blood flow), and is conducive to the passive targeting enrichment of nanoparticles in tumor lesions, this is the basis of active targeting of nanoparticles (Youn & Bae, 2018; Kwon et al., 2019). The size of nanoparticles is the key factor for passive targeting to tumor. The intercellular gap of tumor vascular endothelial cells is in the range of 100–600 nm (Maruyama, 2011). If the diameter of nanoparticles is larger than 100 nm, it will remain in the liver, and if it is less than 100 nm, then it can be excreted by the kidney (Mitragotri & Lahann, 2009). Thus, 10–100 nm nanoparticles are suited for solid tumor targeting. By altering the ratio and reaction conditions of carboxymethyl chitosan and etidronic acid, nanoparticles can be synthesized around 70 nm.

Movahedi labeled anti-MR antibody with radioactive element technetium-99, and observed the targeting effect of antibody at tumor site in the nude mice (Mitragotri & Lahann, 2009). In the present study, we observed rapid accumulation of MIC-NPs at the tumor site and it has been found to remain for a longer time in mice, which confirmed the experiment results of Movahedi, and also showed that MIC-NPs can target not only M2, but also the tumor site effectively (Movahedi et al., 2012).

Cytotoxicity studies *in vitro* and *in vivo* showed that MIC-NPs were safe. As the anti-tumor mechanism of MIC-NPs might be immunological, α-interferon was used as the control drug. Compared with the NS group and interferon group, high, middle and low doses of MIC-NPs exhibited significant anti-tumor activities. The result indicated that MIC-NPs have anti-tumor effect *in vivo*, and there is no relationship between the dose of MIC-NPs and its curative effect.

The isoelectric point of the MIC-NPs is about pH value 7.2; therefore, it is positively charged in a solution above pH 7.2 and negatively charged in the pH below 7.2. In order to make the nanoparticles penetrate into the solid tumor, the cationic nanoparticles are often used, which is based on the negative property of the cell membrane (including vascular endothelial cells and tumor cells) and cationic nanoparticles are easy to adsorb by cells (Fasol et al., 2012). The pH value of TME is generally under 7, and the blood pH value is 7.35–7.45. As the isoelectric point of the MIC-NPs is about 7.2, we speculate that the MIC-NPs with negative charge in the blood is beneficial to maintaining dispersion and stability, and the MIC-NPs with positive charge in TME are easy to stay in the TME.

MIC-NPs can effectively target the tumor site and stay for a long time, and thereby it can exert an anti-tumor effect (which may be due to the high permeability and retention effect of solid tumor). MIC-NPs are passively enriched at the tumor focus; MIC-NPs are positively charged in TME, which makes it easy to fully combine with M2 macrophages. Through MR-mediated mechanism, MIC-NPs can actively target and bind to M2 macrophages, and thereby target the TME. By inducing M2 apoptosis, MIC-NPs change the TME and thus the growth of tumor-bearing mice was inhibited.

## 5. Conclusions

In this study, a kind of nanoparticles MIC-NPs was successfully synthesized and its anti-tumor effect was evaluated both *in vivo* and *in vitro*. The nanoparticles exhibited a spherical morphology and good dispersion property. Toxicity study *in vitro* and *in vivo* indicated that MIC-NPs were safe. *In vitro* cellular uptake and cell growth inhibition experiments demonstrated that MIC-NPs can target inhibition to M2, and its targeted mechanism was mediated by MR. The pharmacokinetic experiments revealed the selective accumulation of MIC-NPs at tumor site. Based on tumor volume measurements and immunological test, MIC-NPs were found to have significantly anti-tumor activity. In summary, MIC-NPs may have potential applications in tumor therapy.

## Figures and Tables

**Scheme 1.**
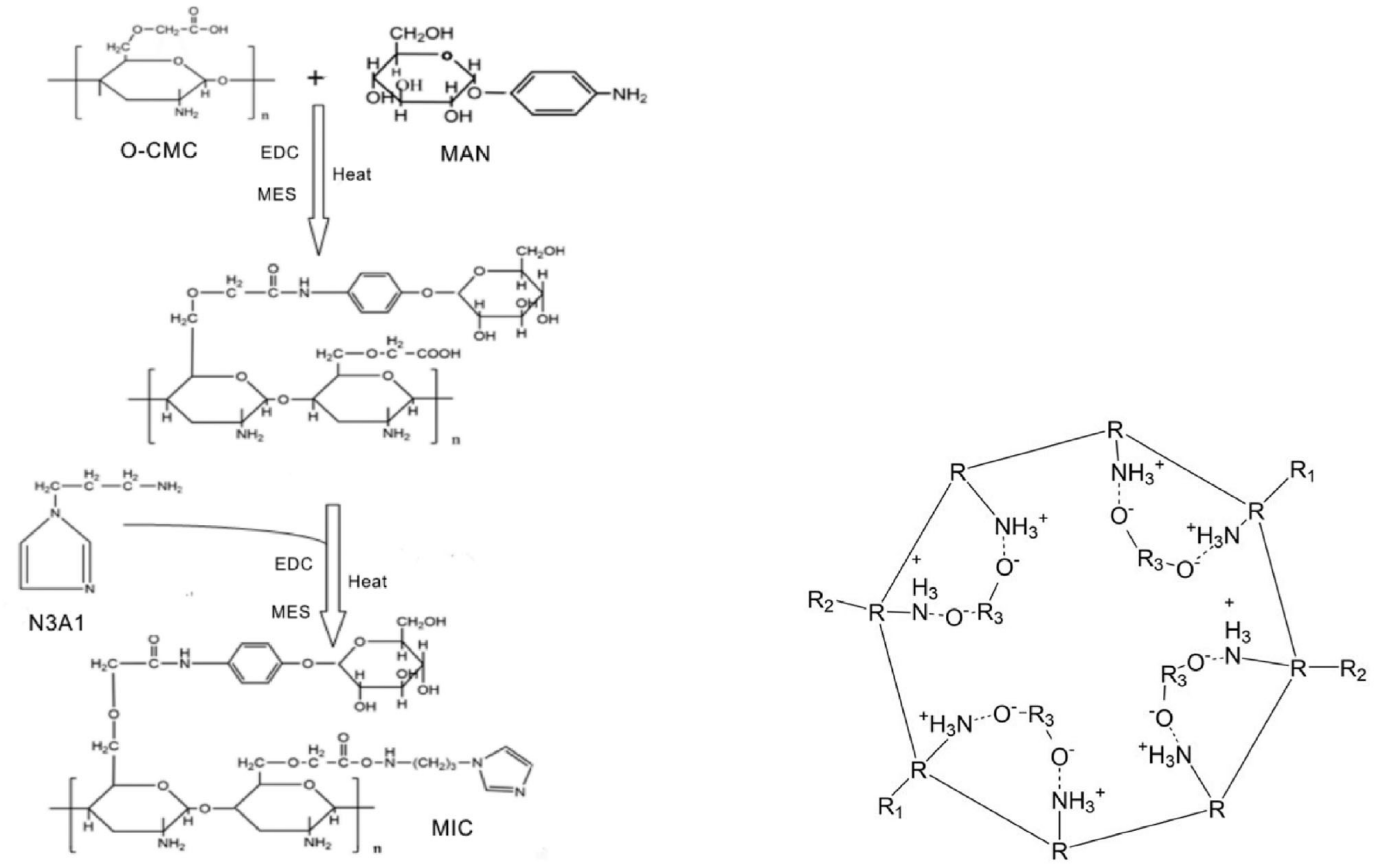
Scheme representing MIC synthesis and chemical structures of MIC-NPs.
